# The Genomic HyperBrowser: an analysis web server for genome-scale data

**DOI:** 10.1093/nar/gkt342

**Published:** 2013-04-30

**Authors:** Geir K. Sandve, Sveinung Gundersen, Morten Johansen, Ingrid K. Glad, Krishanthi Gunathasan, Lars Holden, Marit Holden, Knut Liestøl, Ståle Nygård, Vegard Nygaard, Jonas Paulsen, Halfdan Rydbeck, Kai Trengereid, Trevor Clancy, Finn Drabløs, Egil Ferkingstad, Matúš Kalaš, Tonje Lien, Morten B. Rye, Arnoldo Frigessi, Eivind Hovig

**Affiliations:** ^1^Department of Informatics, University of Oslo, PO Box 1080, Blindern, 0316 Oslo, Norway, ^2^Centre for Cancer Biomedicine, Faculty of Medicine, University of Oslo, PO Box 4950, Nydalen, 0424 Oslo, Norway, ^3^Department of Tumor Biology, Institute for Cancer Research, The Norwegian Radium Hospital, Oslo University Hospital, PO Box 4950 Nydalen, 0424 Oslo, Norway, ^4^Institute for Medical Informatics, The Norwegian Radium Hospital, Oslo University Hospital, PO Box 4950, Nydalen, N-0424 Oslo, Norway, ^5^Department of Mathematics, University of Oslo, PO Box 1053, Blindern, 0316 Oslo, Norway, ^6^Department of Medical Biology, Faculty of Health Science, University of Tromsø, 9037 Tromsø, Norway, ^7^Statistics For Innovation, Norwegian Computing Center, 0314 Oslo, Norway, ^8^Bioinformatics Core Facility, Oslo University Hospital and University of Oslo, PO Box 4950 Nydalen, N-0424 Oslo, Norway, ^9^Department of Cancer Research and Molecular Medicine, Norwegian University of Science and Technology (NTNU), 7491 Trondheim, Norway, ^10^Department of Informatics, University of Bergen, PO Box 7803, 5020 Bergen, Norway, ^11^Computational Biology Unit, Uni Computing, Uni Research AS, 5020 Bergen, Norway and ^12^Department of Biostatistics, Institute of Basic Medical Sciences, University of Oslo, PO Box 1122 Blindern, 0317 Oslo, Norway

## Abstract

The immense increase in availability of genomic scale datasets, such as those provided by the ENCODE and Roadmap Epigenomics projects, presents unprecedented opportunities for individual researchers to pose novel falsifiable biological questions. With this opportunity, however, researchers are faced with the challenge of how to best analyze and interpret their genome-scale datasets. A powerful way of representing genome-scale data is as feature-specific coordinates relative to reference genome assemblies, i.e. as genomic tracks. The Genomic HyperBrowser (http://hyperbrowser.uio.no) is an open-ended web server for the analysis of genomic track data. Through the provision of several highly customizable components for processing and statistical analysis of genomic tracks, the HyperBrowser opens for a range of genomic investigations, related to, e.g., gene regulation, disease association or epigenetic modifications of the genome.

## INTRODUCTION

The immense increase in the production of genomic scale datasets, e.g., through the ENCODE (1) and Roadmap Epigenomics (2) projects, poses an unmet challenge in terms of available methodology and tools for analytic investigations. These datasets provide unprecedented opportunities for individual researchers to elucidate particular biological mechanisms. However, analysis of these datasets and their relations to each other typically require development of a range of *ad hoc* scripts for generating, manipulating and analyzing genomic data.

For a range of organisms, well-established and internationally accepted reference genome assemblies now exist. Using coordinates on such assemblies, data related to particular locations on the genome can be represented in a precise and unambiguous manner. This avoids many previous difficulties in the field, such as confusion due to incompatible gene terminology. A genome-wide collection of coordinates for a particular genomic feature is often referred to as a genome annotation track, or just genomic track. Such genomic tracks can, e.g., refer to the location of genes, binding of transcription factors, methylation of DNA or modification of histones. Genomic tracks not only allow unified visualization and browsing, such as through the UCSC Genome Browser (3), but also provide a powerful and unified basis for statistical analysis. The base pair positions of reference genomes serve as coordinates on a line, allowing entities such as genes or epigenetic modifications to be viewed as elements positioned on such a line. A statistical question, posed on the relation between two genome-scale datasets, may then be formulated as a simple question relating such elements. An example is to ask whether points on a reference line as defined by one dataset falls unexpectedly often within segments on the same line as defined by another dataset.

The Genomic HyperBrowser web server provides a broad suite of functionality for rigorous statistical analysis of genomic data. At the core of the system is a set of statistical analyses, available through a single tool: ‘Analyze genomic tracks’. Descriptive statistics, test statistics and null models are described in terms of well-defined elements along a linear representation of the genome, in the form of genomic tracks. This tool and its underlying methodology has been described in a previous publication (4), and has since been expanded with tens of new descriptive analyses and hypothesis tests. The statistical analysis is augmented by a collection of data preparation tools that support the processing of genomic data into forms that subsequently allow sophisticated questions to be posed in a simple and intuitive manner. All 42 tools at the server are based on the generic treatment of genomic data as elements along a linear representation of the genome, allowing questions related to different biological application domains to be treated in the same manner. The tools share an underlying analysis code base, which is open-source and tightly integrated with the Galaxy framework (5) for handling of web access, users and data. Through the integration with Galaxy, the standard Galaxy tools are also available and can be used together with the HyperBrowser-specific functionality. The HyperBrowser website is free and open to all, and there is no login requirement.

**Figure 1.**
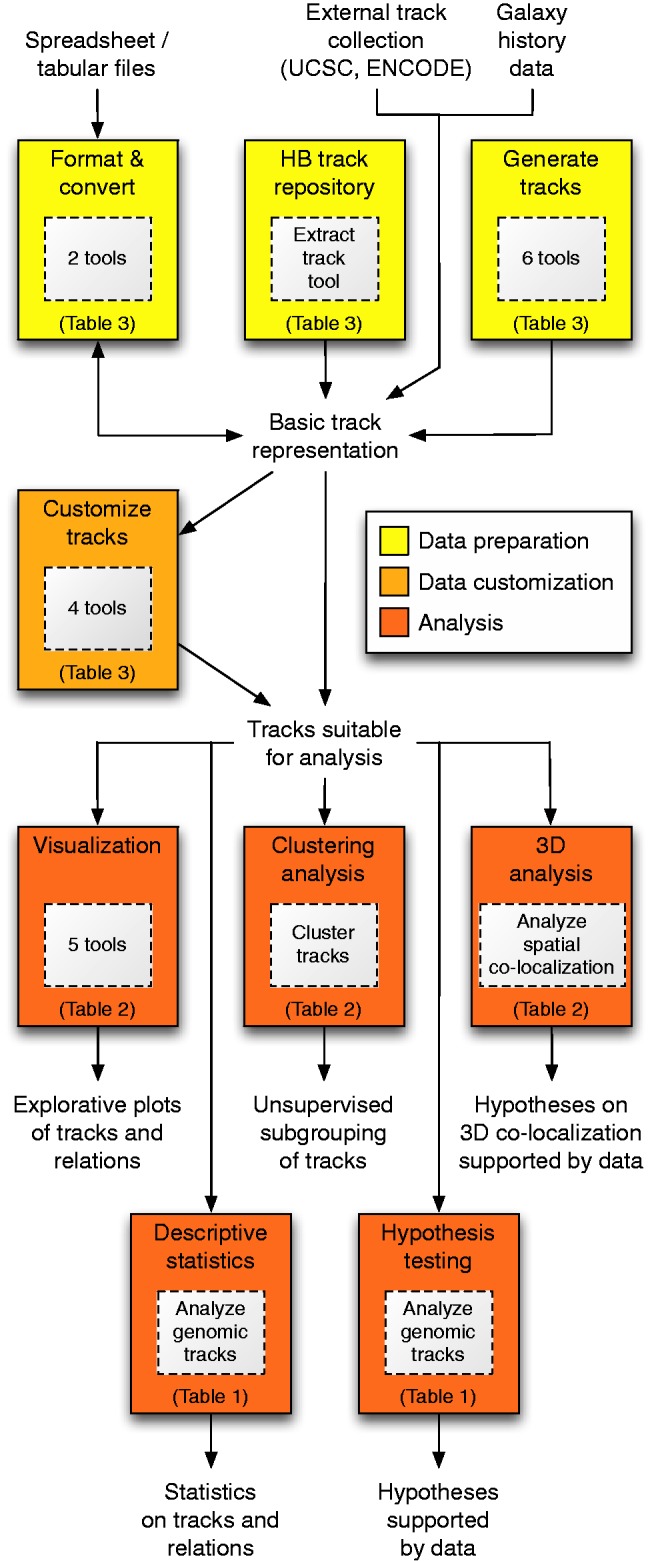
Schematic overview of tool categories available at the Genomic HyperBrowser server. The figure indicates at which points of a typical analysis scenario the various tools may be of use, from the initial collection and preparation of data, through customization of data to match the analysis, to the statistical evaluation of a biological hypothesis. For boxes representing several tools, the precise list of tools can be found under the corresponding header in the table that is referred to (for instance, the two tools represented by the ‘Format and convert’ box can be found under the heading ‘Format and convert tracks’ of Table 3).

The Genomic HyperBrowser is designed to be as open-ended as possible: instead of being developed around a few canonical usage scenarios, it provides a core set of abstractions and components that can be used and combined in a myriad of ways to answer precisely formulated biological questions. Figure 1 gives a schematic overview of how various tools at the HyperBrowser server can be used as part of a full analysis scenario.

## ANALYSIS OF GENOMIC TRACKS

A large collection of analytical functionality is available through the tool ‘Analyze genomic tracks’ under the ‘HyperBrowser analysis’ menu. This opens for a range of genomic investigations that query characteristics of individual tracks or relations between pairs of tracks along the genome (4). After selecting one or a pair of tracks, the analysis of interest can be selected among a set of analyses deemed meaningful based on the type of track(s) selected. For instance, selecting two tracks of segments (intervals) along the genome (e.g. two tracks of ChIP-seq peak regions, without any values associated with the peaks) will allow questions related to co-localization (overlap). On the other hand, selecting two tracks of values per base pair along the genome (e.g. two tracks of bp-level ChIP-seq signal values for every position of the genome) will allow questions related to correlation of values. The HyperBrowser system distinguishes between 15 types of tracks at the generic level (6), where the most widespread types are tracks of points and segments.

**Figure 2.**
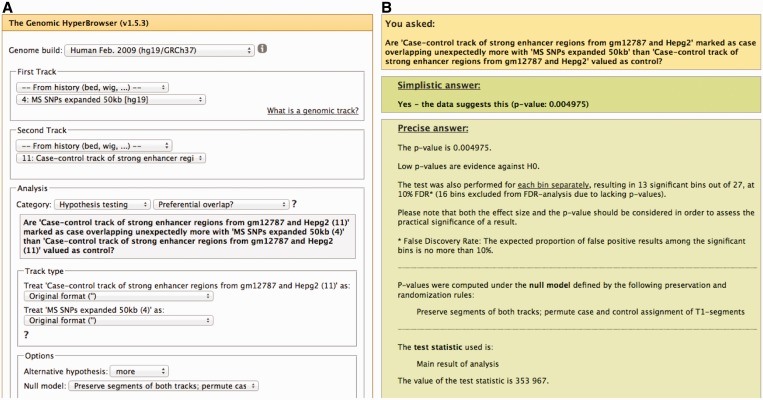
Screenshots of the web interface and results page for the ‘Analyze genomic tracks’ tool. (**A**) Input data, analyses of interest, and analysis parameters are precisely specified through a set of selection boxes. (**B**) The result page provides a main conclusion from the statistical test, as well as a range of details that can be inspected by following various links from the main results page.

Analyses are divided into descriptive statistics (such as counts, base pair coverage and averages) and hypothesis tests (such as whether two tracks are overlapping more than expected by chance). A total of 56 descriptive statistics and 20 hypothesis tests are available, depending on the type of tracks (listed in Table 1). Each hypothesis test may be seen as a generic genomic question that can be parameterized in several ways. The statistical testing procedure used to resolve the question not only varies between questions, but also between parameterizations. One parameterization is the selection of an appropriate null model. Statistical hypothesis testing requires a notion of randomness for the null hypothesis, and careful attention has been given to making such randomness assumptions transparent to the user. For most tests, the randomness assumptions can also be selected from a list of possibly meaningful alternatives (Figure 2A). For instance, one can for hypothesis tests involving a gene track choose a simple null model where genes are randomized independently and uniformly along the genome. Alternatively, one can select a null model where the empirically observed clustering tendency of genes (distribution of inter-gene distances) is preserved. A further alternative is to sample gene positions according to a separately specified intensity track, which can for instance be used to control for influence by external confounders. Depending on the assumptions deemed appropriate by the user for the hypothesis test (through, e.g., the selection of a null model), the system will determine whether to use either an asymptotic computation or a Monte Carlo (MC) based evaluation of *P*-values. This is handled by the system, but at the same time transparent to the user. For MC-based evaluation of *P*-values, a sequential sampling scheme, MCFDR, is used to automatically determine the appropriate number of samples for statistical testing (9).

**Table 1.** Selected descriptive statistics and hypothesis tests available through the ‘Analyze genomic tracks’ tool of the Genomic HyperBrowser

Track1 type	Track2 type	Statistical investigation	Description
Descriptive statistics			
P		Counts	The number of track1-points
P		Frequency	The frequency of track1-points
P		Mean and variance of gaps	Mean and variance of gaps between track1-points
P	P	Frequency proportion	The proportion of all points (track1 and track2) arising from track1
P	P	Point distances	The distribution of distances from each track1-point to the nearest track2-point
P	S	Count inside/outside	The number and proportion of track1-points inside and outside track2-segments
P	S	Matrix of count inside	The number of track1-points inside track2-segments, for all combinations of categories from both tracks
P	S	Relative position within segments	The average relative position of track1-points within track2-segments
P	S	Point to segment distances	The distribution of distances from each track1-point to the nearest track2-segment

S		Bp coverage	The number of base pairs covered by track1
S		Proportional coverage	The proportion of total base pairs covered by track1
S		Avg. segment length	The average length of segments of track1
S		Segment lengths	The distribution of lengths of each track1-segment
S	S	Coverage	Base pair and proportional coverage by track1, track2 and by both
S	S	Enrichment	The enrichment of track1 inside track2 and vice versa, at the bp level
S	S	Segment distances	The distribution of distances from each track1-segment to the nearest track2-segment

F		Mean	The mean value of track1
F		Sum	The sum of values of track1
F		Variance	The variance of values of track1
F		Min and max	The extreme values (min/max) of track1
F	P	Mean at points	The mean value of track1 at positions of track2
F	S	Mean inside and outside	The mean value of track1 inside track2 and outside track2
F	F	CC	Pearson's correlation coefficient of track1 and track2

VP		Values	The distribution of values of track1-elements
VP	S	Values inside	The distribution of values of track1-elements inside track2-elements
VS (c/c)	P	Inside case versus control	The number of track2-points that falls inside track1-segments marked as case or control
VP (c/c)	VS (c/c)	Two-by-two table of inside	Two-by-two table of case/control track1-points that falls inside case/control track2-segments
VS (cat)		Category bp coverage	The number of base pairs covered by each category of track1
VS (cat)		Category point count	The number of elements of each category of track1
VP (cat)	VS (cat)	Contingency table of inside	Contingency table of categorical track1-points that falls inside categorical track2-segments

L		Number of nodes and edges	The number of nodes and edges in track1
L		Number of neighbors	The distribution of the number of neighbors for each node in the graph (track1)
L (w)		Edge weights	The distribution of weights for each edge of the graph (track1)
L (w)		Clustered heatmap of graph	Clustered heatmap of weights of the graph (track1)

Hypothesis tests			

P	P	Different frequencies?	Where is the relative frequency of points of track1 different from the relative frequency of points of track2, more than expected by chance?
P	P	Located nearby?	Are the points of track1 closer to the points of track2 than expected by chance?
P	S	Located inside?	Are the points of track1 falling inside the segments of track2, more than expected by chance?
P	S	Located non-uniformly inside?	Do the points of track1 tend to accumulate more toward the borders of the segments of track2?
P	S	Located nearby?	Are the points of track1 closer to the segments of track2 than expected by chance?

S	S	Similar segments?	Are track1-segments similar (in position and length) to track2-segments, more than expected by chance?
S	S	Overlap?	Are the segments of track1 overlapping the segments of track2, more than expected by chance?
S	S	Located nearby?	Are the segments of track1 closer to the segments of track2 than expected by chance?
F	F	Correlated?	Are the values of track1 and track2 more positively correlated than expected by chance?
P	F	Higher values at locations?	Are the values of track2 higher at the points of track1, than what is expected by chance?
S	F	Higher values inside?	Are the values of track2 higher inside the segments of track1, than what is expected by chance?

P	VS	Located in segments with high values?	Does the number of track1-points that fall in track2-segments depend on the value of track2-segments?
S	VP	Higher values inside segments?	Do the points of track2 that occur inside segments of track1 have higher values than points occurring outside the segments of track1?
VP	VP	Nearby values similar?	When track1-points and track2-point are nearby each other, are the values more similar than expected by chance?
P	VS (c/c)	Located in case segments	Does the number of track1-points that fall in track2-segments depend on whether the track2-segments are marked as case or control?
VS (c/c)	S	Preferential overlap?	Are the segments of track1 marked as case overlapping unexpectedly more with the segments of track2 than the segments of track1 marked as control?
VP (cat)	VS (cat)	Category pairs differentially co-located?	Which categories of track1-points fall more inside which categories of track2-segments?

LGP	P	Co-localized in 3D?	Are the points of track2 closer in 3D (as defined by track1) than expected by chance?

Each analysis is defined for either one or two tracks, with the corresponding track type denoted in the columns ‘Track1 type’ and ‘Track2 type’. The track type abbreviations, as defined in (6), are as follows: Points (P), Segments (S), Valued Points (VP), Valued Segments (VS), Function (F), Linked Genome Partition (LGP) and any Linked (L) track. In addition, attached values are: number (default), case/control (c/c), category (cat) and weighted edges (w). Most hypothesis tests are available in one- and two-sided versions. Looking at, e.g., overlap, the possible alternative hypotheses would then be whether the segments of track1 are overlapping the segments of track2, more, less or differently than expected by chance. Results of the analyses are given both at the global level and for local regions along the genome. A few of the hypothesis tests relating points and/or segments are also available in specific libraries (7,8), but only for certain null models. In addition, these libraries require low-level command-line access, API access or configuration file setup in order to start analyses.

The output of the ‘Analyze genomic tracks’ tool (Figure 2B) presents the main conclusion from the analysis, along with some interpretations and restrictions on its applicability. This main conclusion is complemented by a range of detailed results in the form of tables and figures, provided at both the global level and for local regions along the genome. The tool emphasizes reproducibility by providing rich analysis output, describing the methodologies that have been used, and reporting all parameter settings and data sources. Screencasts, tutorials and demo buttons for five genome analysis examples are provided with the tool.

A set of tools focusing on visual analysis of track data is available under the menu ‘Visual analysis of tracks’. Under the menu ‘Specialized analysis of tracks’, we provide a tool containing a recently developed hypothesis test querying whether the elements of a track are spatially co-localized with respect to the three-dimensional structure of the genome, as defined using results from recent Hi-C experiments (10). A tool for unsupervised analysis of track similarities (clustering) is also available under the same heading (manuscript submitted). Tool details are given in Table 2.

**Table 2.** Tools for statistical, visual and specialized analyses of genomic tracks

Tool name	Description	Genomic example
*Statistical analysis*		
Analyze genomic tracks	The main analysis interface of the Genomic HyperBrowser (4). Executes analyses on a single genomic track or on the relation between two tracks. Allows specification of additional input parameters for the analyses, specifically including the specification of alternative hypotheses and null models for the hypothesis tests. Contains 56 descriptive statistics and 20 hypothesis tests.	Analyze cell-specificity of active chromatin in disease regions, as described in section ‘Full analysis scenario.
*Visual analysis of tracks*		
Visualize track elements relative to anchor regions	Allows visualization of the distribution of track elements along chromosomes, or along custom-specified bins. The specified regions are displayed vertically, in order to simplify visual comparison.	Visualize the detailed positioning of histone modifications relative to the TSS of a selected set of gene regions.
Create high-resolution map of track distribution along genome	Visualizing track elements along a line, such as in the UCSC genome browser or the relative positioning visualization tool, can necessarily only offer a global overview at a very limited resolution. This tool instead uses a fractal layout of the genome line (similar to Hilbert curve (11) to map genome locations to individual pixels in a matrix instead of along a line, effectively increasing the resolution quadratically. Although the interpretation requires a certain effort, this form of visualization can potentially be very informative.	Visualize the genome-wide distribution of a densely populated track, such as repeating elements or a DNase accessibility experiment.
Create high-resolution map of multiple track distributions along genome	Similar to the one-track version above, but uses up to three separate color channels (red,green,blue) to visualize the presence of up to three different tracks in corresponding parts of the genome by combining their color channel values at individual pixels.	Visualize the comparative distribution of DNase accessibility in three different cell types to see patterns of similar and distinct accessibility.
Visualize relation between two tracks across genomic regions	Used to reveal complex relations between tracks along the genome. For each defined analysis region (bin), a score is calculated for both tracks, using the specified summarizing function. The resulting (x,y) scores are then visualized as a single point in a scatter plot.	Plot exon density versus average melting temperature in 10 mbp bins along the genome.
Aggregation plot of track elements relative to anchor regions	Used to reveal trends of how track elements are distributed relative to a set of anchor regions (bins). All anchor regions are divided into the same number of sub-bins, and a summary statistic is calculated for each sub-bin and averaged across all anchor regions. The tool returns a plot of the average values with 95% confidence intervals.	Positions of histone modifications around TSS.
*Specialized analysis of tracks*		
Analyze co-localization of input genomic regions	Analyze a selected track of genome locations for spatial co-localization with respect to the three-dimensional structure of the genome, as defined using results from recent Hi-C experiments. The Hi-C data have been corrected for bias using a method presented in a recent paper (10), and further normalized by sub-tracting the expected signal given the sequential distance between elements.	Analyze whether somatic mutations in cancer are co-localized in 3D in a relevant cell type.
Perform clustering of genomic tracks	Used to investigate relations between multiple tracks in an unsupervised manner (manuscript submitted). This tool allows an essentially unlimited number of tracks to be selected, and further allows the distance measure to be used for the clustering to be precisely specified through selection among a varied set of a notions of track similarity.	Analyze similarities between histone modifications in different cell types.
Analyze k-mer occurrences	Used to analyze a global track of occurrence locations for a specified k-mer from a particular reference genome. All relevant analyses in the ‘Analyze genomic tracks’ tool can be used.	Analyze correlation of a specific k-mer with other tracks, e.g. genes, in order to find functional significance.
Inspect k-mer frequency variation	Used to calculate and visualize the frequency distribution of a particular k-mer along a genome reference. Splits the selected analysis regions (e.g. chromosomes) into a suitable number of subregions (bins). For each bin, the number of occurrences of the selected k-mer is counted and plotted.	Inspect the frequency variation of a particular k-mer along the genome.

Further descriptions are given at the web pages of the tools themselves, along with demo buttons and links to reproducible examples of how each tool can be used. The ‘Analyze genomic tracks’ tool has previously been described (4).

## PROCESSING DATA INTO FORMS SUITABLE FOR ANALYSIS

In many situations, a complex formulation of a biological question may be simplified if the original data are first transformed into a form that more directly reflects the question of interest. An example of this is a question of how often DNA binding locations of a given TF (as a first genomic track) fall inside or in the close vicinity of genes (as a second track). Although clearly manageable, the concept of proximity in this setting requires some thought and further specification. If one transforms the gene track by expanding the gene intervals to include, say, one kbp flanks, one can afterwards ask the more simple question of how often the TF binding locations fall inside these expanded gene intervals. This latter version is easy to envision and does not involve any ambiguity. This example shows the redefinition of a problem originally formulated to involve vicinity to fit with an analysis based on the simpler concept of containment. Thus, by combining a set of basic, generic analyses with a collection of track transformation functionality, a core set of well-understood analyses can be applied to a much broader range of biologically motivated questions. Several tools for customizing data into forms that may simplify subsequent analyses are available under the menu ‘Customize tracks’, and are summarized in Table 3.

**Table 3.** Tools for extracting genomic tracks from the HyperBrowser repository, customizing tracks into forms suitable for a subsequent analysis of interest, generating new tracks, and formatting and converting existing tracks

Tool name	Description	Genomic example
HyperBrowser track repository		
Extract track from HyperBrowser repository	Used to extract datasets from the track repository stored on the HyperBrowser server. Datasets can be extracted in a range of different formats, and from limited regions of the genome, if needed. Also, overlapping segments can be merged.	Extract the RefSeq gene track, in order to expand the gene segments with the ‘Expand BED segments’ tool.
Customize tracks		
Expand BED segments	Allows extracting start-, mid- or endpoints of genomic intervals, as well as expanding either the original intervals or the extracted start-/end-/mid-points. This is useful in a variety of situations where an analysis of interest involves either proximity to or positioning relative to the original track elements, or where a size unification of track elements is desired (based on, e.g., taking midpoints and then expanding a certain distance). Also, if the expanded region crosses any chromosome borders, this is handled correctly.	An example of an analysis involving both proximity and relative positioning is the analysis of histone modification frequencies in bins of particular distances relative to the upstream end points of genes (transcription start sites).
Combine two BED files into single case–control track	Allows combining elements from two separate datasets into a single track where the elements are denoted as case (target) or control, depending on their source. This allows analyses of how other tracks preferentially interact with case elements as opposed to control elements.	An example is to combine chromatin states from two different cell types as case and control elements, in order to ask whether regions associated to MS susceptibility overlap more with case than control segments. See section ‘Full analysis scenario’.
Merge multiple BED files into single categorical track	Allows combining elements from multiple datasets into a single track, denoted with a category that reflects their source.	Merge segment tracks denoting, e.g., exons, introns and intergenic regions in order to create a category track spanning the whole genome.
Generate tracks		
Generate bp-level track from DNA sequence	Supports a rich set of possibilities for constructing tracks based on the DNA sequence itself along a reference genome.	Construct a bp-level track of GC content in a sliding window of selectable size along the genome.
Generate bp-level track of distance to nearest segment	Allows the generation of tracks giving for each bp the distance (in bps) to the nearest element in any track.	Generate a bp-level track of distance to nearest gene.
Generate intensity track for confounder handling	Generates so-called ‘intensity tracks’ which are used in controlling for confounder tracks in particular analyses. The user selects a target track as well as a set of control tracks, i.e. a set of tracks whose influence on the target track one aims to control for. The generated intensity track defines, for each base pair, the probability that an element of the target track lands at that position during randomization. The intensity track can afterwards be selected as part of the null model specification when doing hypothesis testing through the ‘Analyze genomic tracks’ tool.	Can, e.g., be used to control for the influence of gene proximity when analyzing the relation between TF binding locations and active regions in a given cell type.
Generate k-mer occurrence track	Generates a global track of occurrence locations for a specified k-mer on a particular reference genome.	Generate a track of all occurrences of the 8-mer ‘ACGTTGCA’ in the human hg19 genome assembly.
Generate track of genes associated with literature terms (using Coremine)	Generates a track of gene segments along the human genome, where the genes are associated with one or more specified literature terms. The associations are provided by the CoreMine medical database, which is regularly updated with term-gene associations mined from published literature.	Find a set of genes associated with melanoma. Each gene will have an attached *P*-value, denoting the strength of the association.
Format and convert tracks		
Convert between GTrack/BED/ WIG/bedGraph/GFF/ FASTA files	The most commonly used formats for genomic location data are (arguably) the formats BED, BedGraph and WIG defined by the UCSC Genome Browser, as well as the format GFF in various versions. The tool allows converting between these formats, to the degree they are able to represent the same information. The tool also allows converting data to and from the recent GTrack format, which is a recent, unified format that is capable of representing data of any track type, and thus data stemming from any of the other file formats (6).	Convert a GTrack file to the BED format in order to use BED-specific Galaxy tools.
Create GTrack file from unstructured tabular data	The tool allows structuring unformatted tabular data into a GTrack file by specifying the necessary meta-data through simple selection boxes, inferring further properties of the data where possible.	Import virus integration sites of the Human Papilloma Virus (HPV) from an Excel spreadsheet into a GTrack file for further analysis by the ‘Analyze genomic tracks’ tool.

Further descriptions are given at the web pages of the tools themselves, along with demo buttons and links to reproducible examples of how each tool can be used. The GTrack-related tools have previously been described (6).

In some analysis scenarios, a feature of interest is not explicitly available in the form of a genomic track, but can be derived from properties of other genomic tracks. The HyperBrowser menu ‘Generate tracks’ includes several tools for generation of datasets in such situations. Tracks can be generated based on DNA sequence properties along the genome, or based on density of, or distance to, certain genomic features along the genome. An overview of these tools is given in Table 3.

In other analysis scenarios, genomic coordinates are available for the data of interest, but not in a format that can be readily used in the tool of interest. Genomic datasets come in a variety of forms, including raw lists of coordinates not adhering to any specified format. The data are usually in tabular format, typically as raw text files or as spreadsheet documents. The HyperBrowser recognizes most commonly used tabular formats, in addition to a recent unified format, GTrack, supporting all 15 basic types of tracks handled by the system. A format conversion tool is available under the menu ‘Format and convert tracks’, alongside a tool for structuring raw tabular data into a GTrack file (Table 3). A set of tools for validating and editing GTrack files are also available, as introduced in (6).

## SUPPLEMENTING GUI SELECTION WITH COMMAND-BASED BATCH EXECUTION

A web interface based primarily on point-and-click selection has several advantages compared to a command-line-based approach to data analysis. A main advantage is that it does not require the recollection of suitable commands and parameters to achieve a given analysis objective. A typical disadvantage is that it may be cumbersome to perform a multitude of similar analyses. This is in contrast to the command-based approach, where slight modifications to an analysis can often be done very quickly, and where looping may allow multiple analyses to be performed without a huge manual effort. We believe this is rapidly becoming an important issue for genome analysis, as e.g. the ENCODE and Roadmap Epigenomics projects generate chromatin and transcription factor binding tracks for hundreds of different cell types.

To meet this challenge, we have combined advantages of both worlds, the point-and-click based and the command based, through what we refer to as ‘batch execution functionality’. For the initial specification of an analysis, we mainly rely on a GUI-based approach, using selection boxes as described in the section ‘Analysis of genomic tracks’. After an analysis has been specified through the GUI, one can click on ‘Inspect parameters of the analysis’ to obtain a ‘corresponding batch command line’. This purely textual representation of the analysis can now be modified and/or duplicated according to customized needs, and executed in the ‘Execute batch commands’ tool under the menu ‘Text-based analysis interface’. Two options that increase the flexibility is the possibility to use a slash (/) to denote that an analysis is to be performed with multiple alternative tracks or parameter values, and the use of a star character (*) to denote that a given analysis is to be performed on all sub-tracks at a given level of the HyperBrowser track collection hierarchy. These extensions of the format greatly simplify the process of running a given analysis on a set of related tracks, e.g., for different chromatin marks or cell lines.

## FULL ANALYSIS SCENARIO

The full reach of the Genomic HyperBrowser system becomes apparent when considering the combination of various tools for processing and analyzing data. By employing an appropriate combination of data preparation and analysis functionality, a range of sophisticated and precisely specified hypotheses can be investigated.

An example of such an analysis is the investigation of whether regions associated with a given disease overlap preferentially with marks of active chromatin in a certain cell type compared to another reference cell type. A sequence of steps for analyzing multiple sclerosis (MS) associated regions in B-cells versus hepatocytes is given in a Galaxy Page at http://bit.ly/hb_example. This page shows the sequence of tools that has been used, along with the exact input parameters and resulting outputs for each of the tools. Any step can be easily reproduced exactly or with modifications to the input parameters. The analysis starts with a set of SNP coordinates in a form reflecting a typical starting point with data in a raw text or a spreadsheet document. The SNP data are uploaded and formatted, and two genomic tracks of active chromatin state regions (12) in B-cells and hepatocytes are extracted from the HyperBrowser track repository. In their original track representations, the question of interest would be whether the track of active regions in B-cells shows a stronger presence in the vicinity of SNP positions than the hepatocyte track, after appropriate normalization based on overall differences between the tracks of active regions. Both the concept of vicinity and the need for normalization complicates the precise formulation of an appropriate question. By expanding the SNPs to include flanks, and by combining the two tracks of active regions into a single case–control track, the final question becomes whether the MS SNP proximity regions overlap preferentially with segments of the combined active chromatin state track marked as case versus control. As can be seen from the result output of the final step of the analysis, this is indeed the case (13).

The Genomic HyperBrowser is complementarily integrated with other systems for working with genomic track data, both conceptually and implementation-wise. A powerful way to work with genomic data may be to, e.g., first get some general impressions and ideas about the data through direct visualization and browsing in the UCSC genome browser (3), followed by genome-scale exploration using EpiExplorer (14). Relevant hypotheses may then be evaluated by robust statistical analysis within the Genomic HyperBrowser. Throughout such an analysis scenario, one may also use a variety of Galaxy tools that work well together with all the mentioned systems.

## CONCLUSIONS

The Genomic HyperBrowser is a comprehensive system for statistical analysis of genomic tracks. A range of genomic investigations can be addressed through a combination of data processing and analysis tools. Novel features and analyses are continually added to the system. Furthermore, if a user faces a track analysis challenge that cannot be resolved through the present version of the system, we take it upon us to react promptly to expand the system.

## FUNDING

EMBIO, UiO, Helse Sør-Øst, Norwegian Cancer Society, Elixir-Norway, FUGE and eSysbio (the last two are funded by the Research Council of Norway). ‘Statistics for Innovation’, one of the ‘Centers for Research-based Innovation’ funded by the Research Council of Norway. Funding for open access charge: Oslo University Hospital.

*Conflict of interest statement*. Eivind Hovig is a shareholder of PubGene, Inc. All other authors declare that they have no competing interests.
